# Dramatic increases in blood glutamate concentrations are closely related to traumatic brain injury-induced acute lung injury

**DOI:** 10.1038/s41598-017-05574-9

**Published:** 2017-07-14

**Authors:** Wei Bai, Wan-Li Zhu, Ya-Lei Ning, Ping Li, Yan Zhao, Nan Yang, Xing Chen, Yu-Lin Jiang, Wen-Qun Yang, Dong-Po Jiang, Li-Yong Chen, Yuan-Guo Zhou

**Affiliations:** 1 Molecular Biology Center, State Key Laboratory of Trauma, Burn, and Combined Injury, Research Institute of Surgery and Daping Hospital, Third Military Medical University, Chongqing, China; 2 Department of Neurosurgery, Research Institute of Surgery and Daping Hospital, Third Military Medical University, Chongqing, China; 3 Department of ICU, Research Institute of Surgery and Daping Hospital, Third Military Medical University, Chongqing, China; 4 Department of Anesthesiology, Research Institute of Surgery and Daping Hospital, Third Military Medical University, Chongqing, China

## Abstract

Traumatic brain injury-induced acute lung injury (TBI-ALI) is a serious complication after brain injury for which predictive factors are lacking. In this study, we found significantly elevated blood glutamate concentrations in patients with TBI or multiple peripheral trauma (MPT), and patients with more severe injuries showed higher blood glutamate concentrations and longer durations of elevated levels. Although the increase in amplitude was similar between the two groups, the duration was longer in the patients with TBI. There were no significant differences in blood glutamate concentrations in the patients with MPT with regard to ALI status, but the blood glutamate levels were significantly higher in the patients with TBI-ALI than in those without ALI. Moreover, compared to patients without ALI, patients with TBI showed a clearly enhanced inflammatory response that was closely correlated with the blood glutamate levels. The blood glutamate concentration was also found to be a risk factor (adjusted odds ratio, 2.229; 95% CI, 1.082–2.634) and was a better predictor of TBI-ALI than the Glasgow Coma Scale (GCS) score. These results indicated that dramatically increased blood glutamate concentrations were closely related to the occurrence of TBI-ALI and could be used as a predictive marker for “at-risk” patients.

## Introduction

Traumatic brain injury (TBI) is a common, major cause of disability and death that occurs secondarily to accidents, competitive sports and even war. A primary lesion forms at the point of impact, but there are also secondary injuries of TBI, including secondary brain injury and peripheral organ injuries^1, 2^, which are key factors influencing the clinical prognosis^3^. Previous studies have primarily focused on secondary brain injuries caused by rapidly elevated brain glutamate-induced excitotoxicity^4, 5^. However, acute lung injury (ALI) induced by brain injury, which is also known as traumatic brain injury-induced acute lung injury (TBI-ALI) or neurogenic pulmonary oedema (NPE), is another salient cause of patient death^6, 7^. Although it has been reported that the incidence of TBI-ALI might reach 50% or more^8^, this disease has not received sufficient attention in clinical practice because it is relatively unpredictable, has a lack of specific, aetiological diagnostic markers and has similar clinical manifestations to severe pulmonary infection. Thus the diagnosis of TBI-ALI is mainly dependent on exclusion, and it is often overlooked or misdiagnosed^9, 10^. In contrast to TBI-ALI, multiple peripheral trauma (MPT), which refers to multiple peripheral organ damage or tissue trauma that results from a variety of causes without central nervous system injury, and multiple peripheral trauma-induced acute lung injury (MPT-ALI) are easily induced by MPT-associated haemorrhagic shock, fat embolism, disseminated intravascular coagulation (DIC), or other causes^11, 12^.

Two classical theories, blast theory and permeability defect theory, have been proposed and are widely accepted as explanations of the pathogenesis of TBI-ALI^13^. However, our recent studies have shown that in addition to brain glutamate levels, blood glutamate levels increase dramatically after TBI, especially in patients with TBI-ALI^14^. Additionally, a previous report found that increased blood glutamate levels might cause peripheral organ damage^15^, but it remained unclear whether these increased levels were related to the occurrence of TBI-ALI.

Therefore, based on our preliminary findings, the aims of this study were to investigate the relationship between high concentrations of blood glutamate and TBI-ALI and to explore the possible role and the predictive power of blood glutamate in the development of TBI-ALI. We accomplished these goals by collecting more cases of TBI and MPT than in previous studies while taking into account that TBI patients often present with peripheral injury. Thus, MPT/MPT-ALI patients served as a trauma control group to eliminate the interference of peripheral injury in TBI/TBI-ALI patients, thus allowing for further clarification of the role of blood glutamate in this particular type of ALI.

## Results

### Patient characteristics

The patients ranged in age from 19 to 87 years, with an average age of 47 years. The cohort was predominantly male (79.3%) and was more likely to have been involved in traffic accidents (59.8%). In total, 78.3% (n = 72) of the patients underwent a surgical procedure. A total of 37 patients were also diagnosed with ALI, including 21 who were diagnosed with TBI-ALI and 16 who were diagnosed with MPT-ALI. The median hospital stay was 24 or 28 days for the patients with TBI or MPT, respectively, and 5 patients died more than 7 days after admission (Table 1). The detailed patient information is listed in Tables S1–S3; the details of the patients who presented with ALI are listed separately in Table 2. A single sample was obtained from 50 healthy volunteers (age range, 43–67 years; average age, 50 years; 68% men).

**Table 1:** Patient Demographics and Baseline Characteristics (N = 92).

Variables (Units)	TBI (n = 50)	MPT (n = 42)	*p*
Age (yrs)	48 (39–57)	44 (35–56)	0.603
Male, n (%)	43 (86.0)	30 (71.4)	0.125
BMI, kg/m^2^	24.8 (2.1)	23.9 (1.9)	0.311
Major extracranial injury, n (%)	10 (20.0)	42 (100.0)	0.000
Time of injury to hospital, h	6.0 (3.2–9.5)	5.5 (4.3–10.4)	0.076
GCS/AIS-ISS at admission	13(7–15)	14.3 (10.8–21.5)	—
**Admission from, n (%)**			0.000
Neurosurgery	39 (78.0)	16 (38.1)	
ICU	11 (22.0)	26 (61.9)	
**Cause of injury, n (%)**			
Traffic accident	35 (70.0)	20 (47.6)	0.035
Fall	10 (20.0)	10 (23.8)	0.801
Other	5 (10.0)	12 (28.6)	0.031
**Treatment, n (%)**			0.021
Conservative therapy	6 (12.0)	14 (22.6)	
Surgical therapy	44 (88.0)	28 (77.4)	
**Complicated ALI, n (%)**			
TBI-ALI	21 (42.0)	—	—
MPT-ALI	—	16 (38.1)	—
Lung infection	5 (10.0)	2 (4.8)	0.662
Death in hospital, n (%)^a^	3 (6.0)	2 (4.8)	1.000
Length of stay	26 (13–60)	28 (20–51)	0.523

—, not applicable. *p*, difference between the two groups as determined by two-tailed Student’s *t*-tests or nonparametric Mann-Whitney U tests. Continuous variables are expressed as the means (SE) or medians (IQR), and categorical variables are expressed as n (%).

AIS-ISS, Abbreviated Injury Scale-Injury Severity Score; ALI, acute lung injury; BMI, body mass index; GCS, Glasgow Coma Scale; MPT, multiple peripheral trauma; TBI, traumatic brain injury.

^a^Patients who died more than 7 days after admission.

**Table 2:** Clinical Characteristics of Patients with ALI (N = 37).

Variables (Units)	TBI-ALI (N = 21)	MPT-ALI (N = 16)
Age, y	46 (33–55)	41 (30–58)
Male, N (%)	10 (46.7)	10 (62.5)
GCS/AIS-ISS	11 (6–14)	13.8 (10.3–20.9)
PaO_2_ (<60 mmHg), n (%)	13 (61.9)	12 (75.0)
PaCO_2_ (>50 mmHg), n (%)	11 (52.4)	10 (62.5)
Spo_2_ (%)	90 (85–94)	89 (88–95)
PaO_2_/FiO_2_ ratio, n (%)		
200~300	5 (23.8)	4 (25.0)
100~200	7 (33.3)	6 (37.5)
<100	9 (42.9)	6 (37.5)
PAWP	12 (8–15)	11 (7–13)
Chest imaging (X-ray/CT)	21/18	16/12
Diagnosis after admission, n (%)		
Within 24 h	15 (71.4)	9 (56.3)
24–48 h	6 (28.6)	7 (43.7)
PEEP (cmH_2_O)	8 (6–12)	7 (5–10)
Length of ventilation, d	12 (9–16)	9 (7–13)
Length of hospital stay, d	29 (21–45)	25 (23–39)
Death in hospital, n (%)	3 (14.3)	2 (12.5)

Data are presented as numbers (%) or medians (interquartile range). AIS-ISS, Abbreviated Injury Scale-Injury Severity Score; ALI, acute lung injury; GCS, Glasgow Coma Scale; MPT, multiple peripheral trauma; PAWP, pulmonary artery wedge pressure; PEEP, positive end-expiratory pressure; TBI, traumatic brain injury.

### Blood glutamate concentration was obviously elevated in patients with TBI or MPT

**Figure 1:**
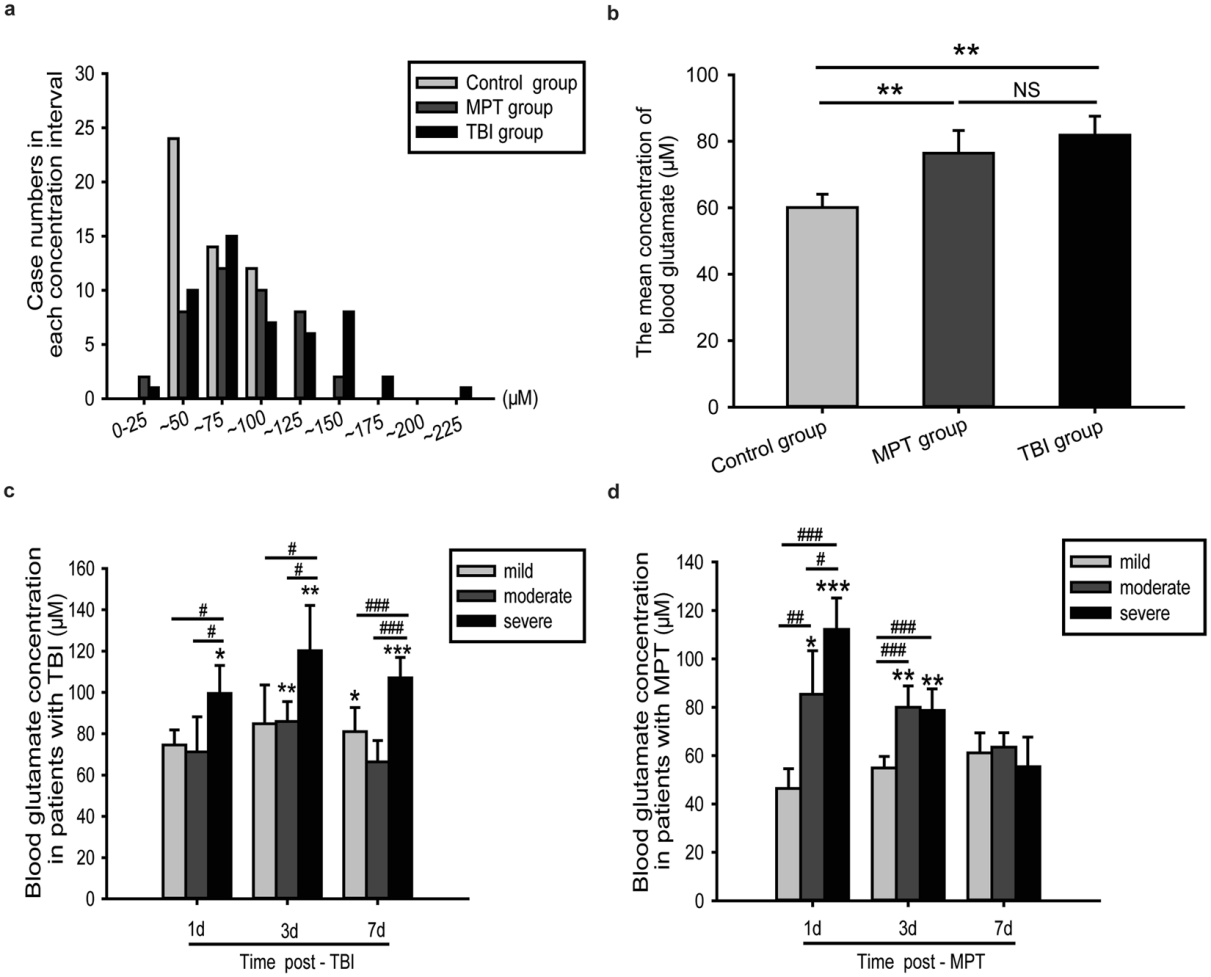
Distribution of patients at each concentration interval (every 25 μM) (**a**) and the mean blood glutamate concentrations (**b**) in the control, MPT and TBI groups. The time-dependent changes in blood glutamate concentration in patients with different severities of TBI (**c**) or MPT **(d**) are shown. *p < 0.05, **p < 0.01, and ***p < 0.001 compared to the control group; ^#^ *p* < 0.05, ^##^ *p* < 0.01, and ^###^ *p* < 0.001 compared between the two groups; NS, not significant. Data are expressed as the means ± SE. Significance was determined by ANOVA with Tukey-Kramer *post hoc* tests or nonparametric Mann-Whitney U tests. MPT, multiple peripheral trauma; TBI, traumatic brain injury.

As shown in Fig. 1a and b, the mean blood glutamate concentration in patients with either TBI or MPT was significantly higher than in the controls (*p* = 0.008 and 0.004, respectively). However, there was no significant difference between the two patient groups (*p* > 0.05) (Fig. 1b). Moreover, we observed time-dependent changes in the blood glutamate concentrations in these two groups. The glutamate concentrations were significantly higher in the patients with TBI than in the controls at 1, 3, and 7 days after admission, and no significant difference was observed among these three time points (Figure S1). However, elevated blood glutamate concentrations were only observed on days 1 and 3 in the patients with MPT, and these levels quickly returned to baseline within 7 days (Figure S1).

Considering that different severities of TBI might result in various elevations of the blood glutamate concentration, we further analysed the relationship between disease severity and glutamate concentration. In the TBI patients, the blood glutamate concentrations significantly increased in those patients with severe injuries within 7 days and were consistently higher than the concentrations in patients with moderate or mild injuries. There was no significant difference between the latter two groups (Figs 1c and S1). However, elevated blood glutamate levels were only observed on the 1^st^ and 3^rd^ day in the patients with MPT (Fig. 1d), although higher blood glutamate concentrations were observed in patients with more severe injuries (Figure S1).

### Elevated blood glutamate concentrations were closely related to the occurrence of TBI-ALI but not MPT-ALI

**Figure 2:**
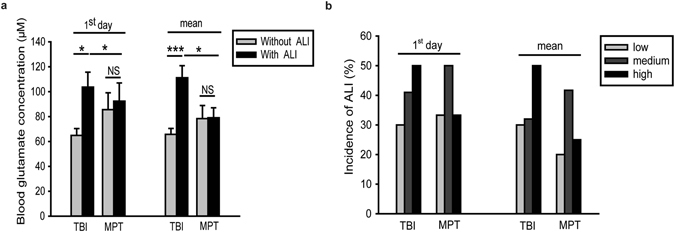
(**a**) Comparison of glutamate concentrations between subgroups (with or without ALI) of patients with TBI or MPT. Data are expressed as the means ± SE. (**b**) Incidence of ALI according to different blood glutamate levels in patients with TBI or MPT. *p < 0.05 and ***p < 0.001 for comparisons between the two groups using two-tailed Student’s *t*-tests or nonparametric Mann-Whitney U tests; NS, not significant. MPT, multiple peripheral trauma; TBI, traumatic brain injury.

To analyse the relationship between blood glutamate concentration and ALI, we further divided the two patient groups into two subgroups according to whether the patient had ALI complications. In both situations, blood glutamate levels were always significantly higher in patients with TBI-ALI than in patients with TBI but without ALI (*p* < 0.001); however, in the MPT group, there was no significant difference in blood glutamate levels between patients with or without ALI (Fig. 2a). For all patients, blood glutamate concentrations were categorised according to the interquartile range (IQR), and the incidence of TBI-ALI in the high-level group was much higher than that in the medium- and low-level groups (*p* = 0.001); however, this trend was not observed in patients with MPT-ALI (Fig. 2b). Additionally, regression analysis showed that blood glutamate concentration upon admission was a risk factor for TBI-ALI (adjusted odds ratio (OR), 2.229; 95% CI, 1.082–2.634; *p* = 0.005) but not for MPT-ALI (adjusted OR, 0.996; 95% CI, 0.965–1.028; *p* = 0.802) (Table 3). Based on these results, we further hypothesised that blood glutamate concentrations might be closely related to the occurrence of TBI-ALI, but not MPT-ALI.

**Table 3:** TBI-ALI Outcome in Relation to Baseline and Treatment Characteristics of Patients with TBI.

Characteristics	OR (95% CI)	*p*	OR (95% CI)	*p*
	unadjusted		adjusted ^**a**^	
Age (yrs)	0.992 (0.959–1.027)	0.653	—	—
Male sex	0.800 (0.169–3.793)	0.779	—	—
BMI, kg/m^2^	1.001 (0.556–2.161)	0.543	—	—
ITH, h	0.782 (0.401–1.752)	0.861	—	—
Major extracranial injury^**b**^	0.934 (0.370–1.483)	0.560		
Admission departments	0.940 (0.277–2.421)	0.905	—	—
Cause of injury	0.608 (0.314–0.891)	0.553	—	—
Treatment				
Blood transfusion	0.597 (0.371–1.418)	0.792	—	—
Ventilation	0.960 (0.841–1.170)	0.893		
Surgical treatment	0.719 (0.311–2.011)	0.831	—	—
Marshall CT score	1.194 (0. 643–1.498)	0.064	0. 819 (0.440–1.105)	0.091
GCS at admission	0.868 (0.745–1.011)	0.068	1.197 (0.981–1.459)	0.076
Blood glutamate^**c**^	4. 301 (1.565–11.825)	0.005	2.229 (1.082–2.634)	0.005

ALI, acute lung injury; BMI, body mass index; GCS, Glasgow Coma Scale; ITH, time of injury to hospital; TBI, traumatic brain injury.

^a^Adjusted for age, sex, BMI, ITH, major extracranial injury, admission departments, cause of injury and treatment measures.

^b^The injury score was calculated according to the number of injured regions.

^c^Represents the glutamate level on admission.

### Blood glutamate levels were closely associated with the inflammatory response in TBI-ALI

In the TBI group, all blood inflammatory markers (procalcitonin (PCT), C-reactive protein (CRP) and counts of white blood cells (WBCs) and neutrophils (NEUs)) were present at higher levels in the patients with ALI than in those without ALI (*p* < 0.05); however, no such difference was observed in the MPT group. Surprisingly, the patients with MPT-ALI had lower CRP levels than the patients without ALI (Table 4). Notably, these results were consistent with the blood glutamate concentrations (Fig. 2a). Concurrently, we found an obvious correlation between blood glutamate levels and inflammatory markers in patients with TBI-ALI (*r *= 0.593, 0.670, 0.659, 0.596 for PCT, CRP, WBCs and NEUs, respectively; *p* = 0.000) (Figure S2). We also noted that, although the intensity of inflammatory responses was similar between groups (Table 4), the patients with TBI-ALI had significantly higher blood glutamate concentrations than the patients with MPT-ALI (*p* = 0.018 for 1^st^ day; *p* < 0.001 for the mean value) (Fig. 2a). These results further suggested that the blood glutamate concentration is closely related to TBI-ALI and might be associated with the inflammatory response in this condition.

**Table 4:** Comparison of Inflammatory Markers in Patients with TBI or MPT.

Variables (Units)^a^	TBI			MPT		
	With	Without	*p*	With	Without	*p*
PCT (ng/mL)	0.24 (0.06–0.52)	0.16 (0.05–0.28)	0.018	1.25 (0.40–4.11)	2.36 (0.29–3.14)	0.997
CRP (mg/L)	62.60 (17.60–99.00)	22.90 (5.60–73.00)	0.024	70 (13.20–92.00)	111.00 (71.00–160.00)	0.030
WBCs count (^10^9^ cells)	11.58 (8.59–13.02)	8.10 (6.41–9.90)	0.002	11.92 (10.37–12.85)	10.92 (8.92–19.34)	0.644
NEUs count (^10^9^ cells)	9.40 (6.70–10.90)	6.24 (4.11–7.52)	0.002	9.64 (9.38–12.28)	8.74 (6.75–18.12)	0.751

*p*, differences between subgroups with or without acute lung injury (ALI) as determined by nonparametric Mann-Whitney U tests. Continuous variables are expressed as medians (IQRs).

CRP, C-reactive protein; MPT, multiple peripheral trauma; NEUs, neutrophils; PCT, procalcitonin; TBI, traumatic brain injury; WBCs, white blood cells.

^a^Represents the mean values at different time points.

### The initial blood glutamate concentration was a better predictor of TBI-ALI than the Glasgow Coma Scale score

**Figure 3:**
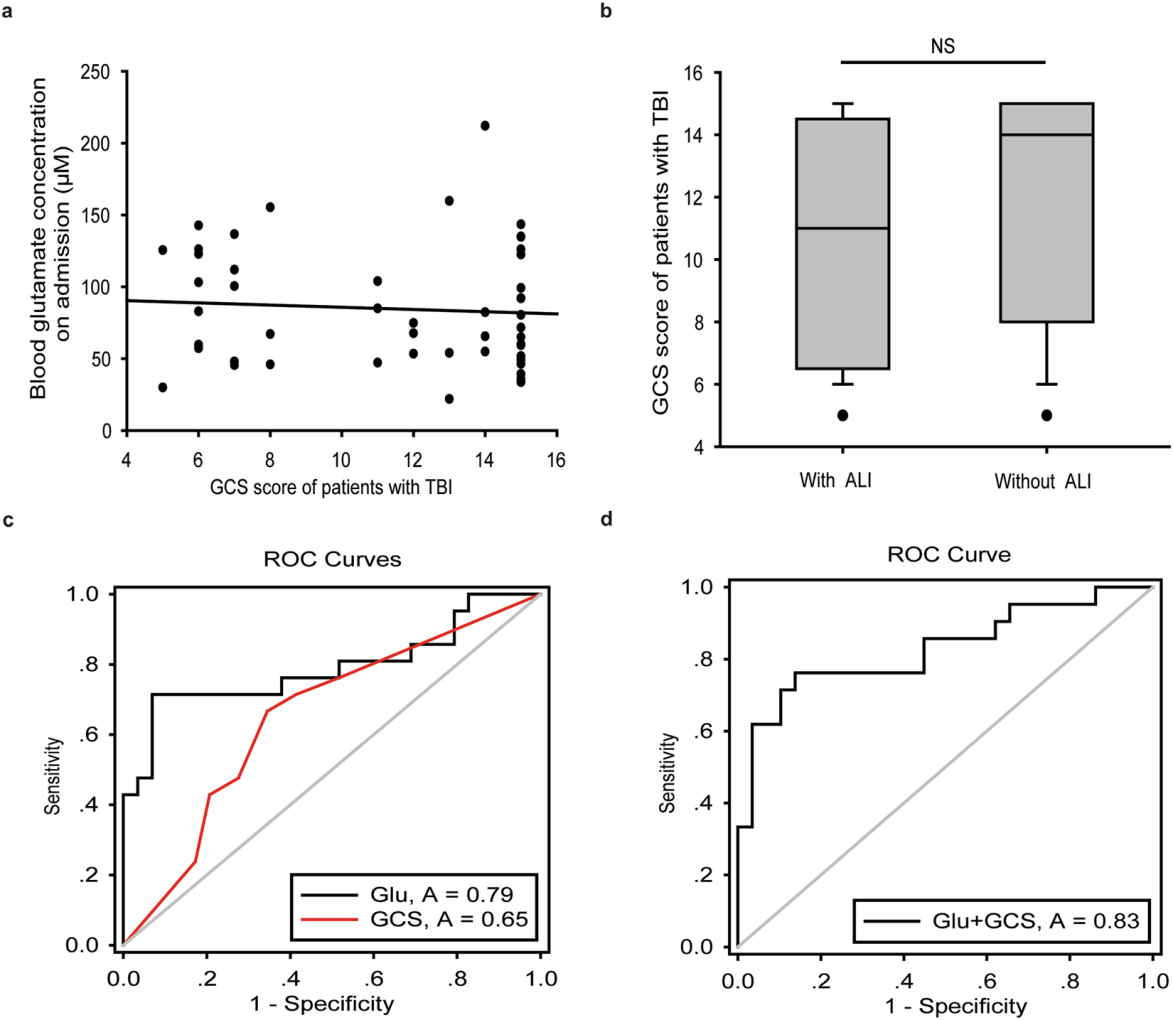
(**a**) There was no significant correlation between blood glutamate levels on admission and GCS score in patients with TBI (*r* = 0.224, *p* = 0.154). (**b**) No significant difference in GCS score was observed between patients with or without TBI-ALI (*p* = 0.062) using nonparametric Mann-Whitney U tests. (**c**). The AUC-ROCs showed that the predictive value of glutamate levels (AUC = 0.792; 95% CI, 0.710–0.873) was better than that of GCS score (AUC = 0.652; 95% CI, 0.565–0.739) (*p* = 0.022) (**d**). ROC curve showing the logistic regression model for the combination of glutamate levels and GCS score (AUC = 0.829; 95% CI, 0.706–0.953), which was better than GCS score (*p* = 0.039) but not glutamate concentration (*p* = 0.243). ALI, acute lung injury; GCS, Glasgow Coma Scale; Glu, glutamate; TBI, traumatic brain injury.

Because the occurrence of TBI-ALI is typically concealed but progresses rapidly (Table 2), we tested whether the Glasgow Coma Scale (GCS) score and the blood glutamate concentration could be used as predictors of patients with TBI within 24 hours of hospital admission. Although the differences in the incidence of TBI-ALI in patients with severe, moderate and mild brain injuries were significant (*p* = 0.002), regression analysis showed that the GCS score was not a risk factor for TBI-ALI (adjusted OR, 1.197; 95% CI, 0.981–1.459; *p* = 0.076) (Table 3). Furthermore, a correlation analysis showed no obvious correlation between the initial blood glutamate levels and the GCS score (*r* = 0.224, *p* = 0.154) (Fig. 3a), and the median GCS score was not higher in patients with ALI than in patients without ALI (*p* = 0.062) (Fig. 3b).

Based on the receiver operating characteristic (ROC) curve, the optimal cut-off value for the blood glutamate level at admission as an indicator for a diagnosis of TBI-ALI was 99.89 μM, which yielded an area under the receiver-operating characteristic curve (AUC-ROC) of 0.792 (95% CI, 0.710–0.873). For the GCS score, the AUC-ROC was 0.652 (95% CI, 0.565–0.739) (Fig. 3c). The likelihood ratio test showed a significant increase in the predictive value of the glutamate concentration compared with that of the GCS score (*p* = 0.022). A logistic regression resulted in a combined glutamate and GCS score model with an AUC-ROC of 0.829 (95% CI, 0.706–0.953). The predictive power of the combined model was better than the GCS score (*p* = 0.039), but not the blood glutamate level alone (*p* = 0.243) (Fig. 3d).

## Discussion

Despite biological variations, blood glutamate concentrations are relatively stable under physiological conditions^16, 17^. However, in the present study, we found that the blood glutamate concentrations of patients with TBI or MPT were significantly higher than those in healthy volunteers and were related to injury severity in both patient groups, although the mechanisms involved may be different. In the patients with TBI, the elevated glutamate concentrations persisted for 7 days, whereas the patients with MPT showed a peak in blood glutamate levels on day 1 that quickly declined to baseline within 7 days. Although the mean glutamate levels were similar between the two patient groups, a subgroup analysis showed that the patients with TBI-ALI had much higher blood glutamate levels than the patients with MPT-ALI, and both subgroups had similar inflammatory response levels. Though the source of blood glutamate remains unclear, previous research found that blood cells, especially neutrophils^18^ and platelets^19^, and peripheral tissues^20^ might be the primary source of blood glutamate. However, considering that the blood-brain barrier (BBB) is effectively “opened twice” after brain injury^21^, the much higher concentrations of intracranial glutamate may contribute substantially to the prolonged increase in blood glutamate concentrations in patients with TBI^22^. By contrast, in patients with MPT, when the inflammatory response is attenuated and the tissue recovers from injury, the level of glutamate released into the blood might rapidly decrease.

TBI-ALI is the major complication following TBI, but its specific pathogenesis remains unclear^10, 23^. Our previous animal experiments revealed that the pathology of TBI-ALI may be related to blood glutamate levels^14^. In the present study, we observed that blood glutamate levels do not correlate with MPT-ALI but are closely related to the occurrence of TBI-ALI, which further confirmed our findings. These results also suggest that ALI alone is not a primary factor for elevated blood glutamate levels. In contrast, in our recent animal studies, blood glutamate did not play an important role in the development of MPT-ALI even if the glutamate levels have reached or exceeded those measured in TBI-ALI (data not shown).

The inflammatory response is the main pathological change during the development of ALI^24^, and previous studies have also found that glutamate signalling plays an important role in lung inflammation^25–27^. In this study, we found a close connection between the levels of blood glutamate and the inflammatory response in patients with TBI-ALI. However, we could not determine whether the elevations in blood glutamate levels were induced by or caused an inflammatory response or even whether there was a reciprocal causal relationship. Our previous research has shown that localised increases in glutamate levels could convert the anti-inflammatory response of adenosine 2A receptor (A_2A_R) to a pro-inflammatory response in the brain^28^. Thus, the polymerisation of metabotropic glutamate receptor 5 (mGluR5) and A_2A_R, which is activated by a high concentration of blood glutamate, is likely to further strengthen this pro-inflammatory effect^14^. This mechanism might offer a possible explanation for this finding. Recent studies have also highlighted the important role of neuroimmunologic modulation in ALI^29^, though the specific mechanism by which blood glutamate contributes to the occurrence and development of TBI-ALI remains unknown and requires further investigation.

It is common in clinical practice to use scores (such as the GCS score) as indicators or references for assessing patient prognoses. However, in this study, the GCS score was less accurate than the blood glutamate level in predicting TBI-ALI. Because the clinical manifestation of TBI-ALI lacks specificity and is often obscured by the primary disease, there is an urgent need for an objective and easily quantifiable parameter to predict the disease; therefore, blood glutamate levels make sense for use in clinical practice. Additionally, several studies have confirmed that lowering blood glutamate levels by haemofiltration^30^ or peritoneal dialysis^31^ exerts a protective effect on the brain. Based on the evidence revealed in this study, we believe that in the future, blood glutamate levels might be a good therapeutic target not only for brain injury but also for complicated lung injury in clinical practice.

### Strengths and limitations

To the best of our knowledge, our study is the first to provide clinical evidence that elevated blood glutamate concentrations are correlated with the incidence of TBI-ALI. We also preliminarily discussed the possible mechanism underlying this relationship and suggested that the blood glutamate concentration could be used to identify and treat at-risk patients. However, because this study was observational in nature rather than a randomised control study, we could not identify the causal relationships between blood glutamate levels, inflammation and TBI-ALI. It is notable, however, that our previous research^14^ and recent animal studies (data not shown) have strongly suggested the triggering role of blood glutamate in TBI-ALI. Therefore, in future studies, it will be necessary to assess these relationships by examining how changes in blood glutamate levels in patients affect the development of ALI and by observing subsequent changes. Another limitation is shown in Table S3: specifically, aspartate transaminase (AST) and alanine aminotransferase (ALT) levels were significantly higher in the patients with MPT. As both of these proteins function as primary blood glutamate-scavenging enzymes, it is unknown whether this elevation was specific; a larger cohort of MPT patients may be needed for this determination. Finally, the relatively small sample size in this study precluded a more refined subgroup classification; therefore, even though the multiple logistic regression analysis was carried out on some major confounding factors (Table 3), there were many other hidden factors involved in this analysis. Thus, our current results must be carefully interpreted, and further studies with a larger sample size study and longer-term outcomes are needed to confirm our findings.

## Conclusions

Blood glutamate concentrations dramatically increased following TBI and were closely related to the inflammatory response in TBI-ALI. Moreover, blood glutamate was found to be a good predictor of TBI-ALI and is a potential target for intervention during this condition.

## Methods

### Patients and controls

From August 2015 to June 2016, 92 hospitalised patients, including 50 diagnosed with TBI and 42 diagnosed with MPT, were recruited from the Neurosurgery and ICU Departments of Daping Hospital after admission. Clinical diagnosis of TBI and indicators for acute head injury were assessed according to National Institute of Health and Care Excellence (NICE) criteria, while MPT was defined as an Abbreviated Injury Scale (AIS) score greater than or equal to 3 in one or more body regions other than the head^32^; all diagnoses were confirmed by computed tomography (CT) or X-ray, and Marshall scores were also recorded^33^. The present study assessed only patients who were older than 16 years. Patients with any significant extracranial injury (AIS > 1), a blast-induced or penetrating head injury, or pre-existing chronic brain diseases (e.g., epilepsy or chronic subdural haematoma) were excluded from the TBI group. Other exclusion criteria for both groups included direct chest trauma or pulmonary contusion, pre-existing major diseases of the lung (e.g., pneumonia or chronic obstructive pulmonary disease (COPD)), liver (e.g., liver dysfunction), heart (e.g., coronary heart disease) or blood system (e.g., haemolytic anaemia)^34, 35^. Additionally, fifty healthy volunteers were selected as normal controls. After admission, the protocols used for treatment of the patients with TBI were based on international guidelines established by the Brain Trauma Foundation (BTF)^36^. The patients with MPT were managed using routine surgical or medical treatments. This study was approved by the ethics committee of the Research Institute of Surgery and Daping Hospital. All participants (or legal guardians) in this study provided written informed consent. All analyses and data handling were performed under the guidelines of the NIH and in accordance with the Declaration of Helsinki and its later amendments. Trial registration: http://www.chictr.org.cn (registered on 19 July, 2015). Unique identifier: ChiCTR-RPC-15006770.

### Injury severity classification

Either the initial GCS score or the Abbreviated Injury Scale-Injury Severity Score (AIS-ISS) was obtained and recorded as appropriate by experienced staff at the time of patient arrival with or without resuscitation after injury. For the patients with TBI, the GCS scores for severe, moderate, and mild cases were 3–7, 8–12, and 13–15 points, respectively^37, 38^. Under the AIS-ISS, the patients with MPT were classified as mild (<16), moderate (16–25) or severe (>25)^39, 40^.

### Diagnosis of complicated ALI

In this study, two types of ALI, TBI-ALI and MPT-ALI, were discussed. Patients meeting The Berlin Definition for ALI/ARDS were characterised by the following: rapidly progressing dyspnoea, cyanosis and other symptoms of respiratory failure, with varying decreases in PaO_2_ and increases in PaCO_2_ after admission; an oxygenation index (PaO_2_/FiO_2_) < 300 with pulmonary arterial wedge pressure (PAWP) < 18 mmHg; chest radiography (CT or X-ray) showing localised or diffused oedema or inflammatory changes; and with or without significantly increased blood inflammatory markers^24, 41^. To exclude interference from concurrent lung infection, we excluded those patients with confirmed pathogenic microbial infections^42^.

### Clinical and laboratory assays

Venous blood samples (5 mL) were collected in heparinised tubes from the hospitalised patients and controls at the time of admission (examination) and at 6:00 am on the 3^rd^ and 7^th^ days after admission. Inflammatory markers (including PCT, CRP, and routine blood markers), indicators of hepatic function (e.g., AST, ALT and albumin) and indicators of renal function (e.g., creatinine and blood urea nitrogen (BUN)) were tested at the clinical laboratory, and the blood glutamate levels were analysed with our previously reported method^43^ at the Molecular Biology Center of our hospital by technicians blinded to the patient groups.

### Statistical analysis

Data are expressed as the means ± standard errors (SE) in histograms and as the means (SE), medians (IQR) and n (%) in tables. Pearson’s chi-squared test was used to analyse categorical variables, and one-way analysis of variance (ANOVA) with Tukey-Kramer’s *post hoc* tests, two-tailed *t*-tests or nonparametric Mann-Whitney U tests were used to analyse continuous variables. Spearman’s correlation test and uni-/multivariate regression models were used to analyse the relationships between two groups; the results were reported as either the *r* value or the unadjusted/adjusted ORs with 95% confidence interval (CI), respectively. The AUC-ROCs were calculated to predict the occurrence of TBI-ALI^44^. A two-tailed value of *p* < 0.05 was considered statistically significant. Statistical analyses were performed using Sigma plot software (version 12.5, Systat Software Inc, San Jose, CA) by a statistician blinded to the study.

### Data availability statement

All data generated or analysed during this study are included in this published article (and its Supplementary files).

## Electronic supplementary material

Dramatic increases in blood glutamate concentrations are closely related to traumatic brain injury-induced acute lung injury
