# Supplementary material for: Dramatic increases in blood glutamate concentrations are closely related to traumatic brain injury-induced acute lung injury
Source: Sci Rep. 2017 Jul 14;7:5380. doi: 10.1038/s41598-017-05574-9 (PMC5511144; doi:10.1038/s41598-017-05574-9)
Supplement: Supplementary file 1 — Dramatic increases in blood glutamate concentrations are closely related to traumatic brain injury-induced acute lung injury [file 41598_2017_5574_MOESM1_ESM.pdf]

Supplementary Materials

Dramatic increases in blood glutamate concentrations are closely related to traumatic brain injury-induced acute lung injury

Authors:

Wei Bai[#]; Wan-Li Zhu[#]; Ya-Lei Ning; Ping Li; Yan Zhao; Nan Yang; Xing Chen; Yu-Lin Jiang; Wen-Qun Yang; Dong-Po Jiang; Li-Yong Chen* and Yuan-Guo Zhou*

# Drs. Bai and Zhu contributed equally to this work.

* For information regarding this article, please address correspondence to Dr. Zhou, MD, PhD (E-mail: zhourick@hotmail.com; ygzhou@tmmu.edu.cn) or Dr. Chen, MD, PhD (E-mail: mzkcly@aliyun.com).

Table S1. Detailed Information on Injuries and Treatments in Patients with TBI (N=50)

Variables	N (%)
**Intracranial lesion**	
Epidural haematoma	5 (10.0)
Subdural haematoma	18 (36.0)
Traumatic SAH	18 (36.0)
Intraparenchymal lesion	36 (72.0)
Skull fracture	20 (40.0)
**Marshall initial CT score**	
Marshall Ⅰ-Ⅱ	22 (44.0)
Marshall Ⅲ-Ⅳ	16 (32.0)
Marshall Ⅴ-Ⅵ	12 (24.0)
**GCS score at admission**	
13-15	24 (48.0)
8-12	11 (22.0)
3-7	15 (30.0)
**Extracranial lesion**	
Abdominal injury	5 (10.0)
Multiple fracture	6 (12.0)
Other injuries	4 (0.08)
**Neurosurgical procedures**	
None	6 (12.0)
Craniotomy	18 (36.0)
Decompressive craniectomy	26 (52.0)

GCS, Glasgow Coma Scale; SAH, subarachnoid haemorrhage; TBI, traumatic brain injury.

Table S2. Detailed Information on Injuries and Treatments in Patients with MPT (N=42)

Variables	N (%)
**Peripheral lesion**	
Abdominal injury	14 (33.3)
Multiple fracture	28 (66.7)
Other injuries	12 (28.6)
**AIS-ISS at admission**	
<16	10 (23.8)
16-25	18 (42.9)
>25	14 (33.3)
**Treatment**	
General treatment	14 (33.3)
Closed thoracic drainage	10 (23.8)
Reduction and fixation	28 (66.7)

AIS-ISS, Abbreviated Injury Scale-Injury Severity Score; MPT, multiple peripheral trauma.

Table S3. Comparison of Renal and Liver Function Between Patients with TBI and those with MPT

Variables (Units)	TBI	MPT	*p*
**Liver function**			
AST (U/L)	29.90 (17.48-50.85)	58.60 (27.90-66.90)	0.011
ALT (U/L)	30.30 (17.40-46.80)	42.90 (24.00-105.00)	0.026
Albumin (g/L)	41.60 (37.31-45.20)	40.5 (38.23-47.01)	0.420
**Renal function**			
Creatinine (mg/L)	66.5 (53.28-78.25)	65.90 (46.78-84.93)	0.969
BUN (g/L)	4.99 (3.67-6.70)	4.93 (3.77-6.80)	0.957

p, difference between the two groups as indicated by nonparametric Mann-Whitney U tests. ALT, alanine aminotransferase; AST, aspartate transaminase; BUN, blood urea nitrogen; MPT, multiple peripheral trauma; TBI, traumatic brain injury.

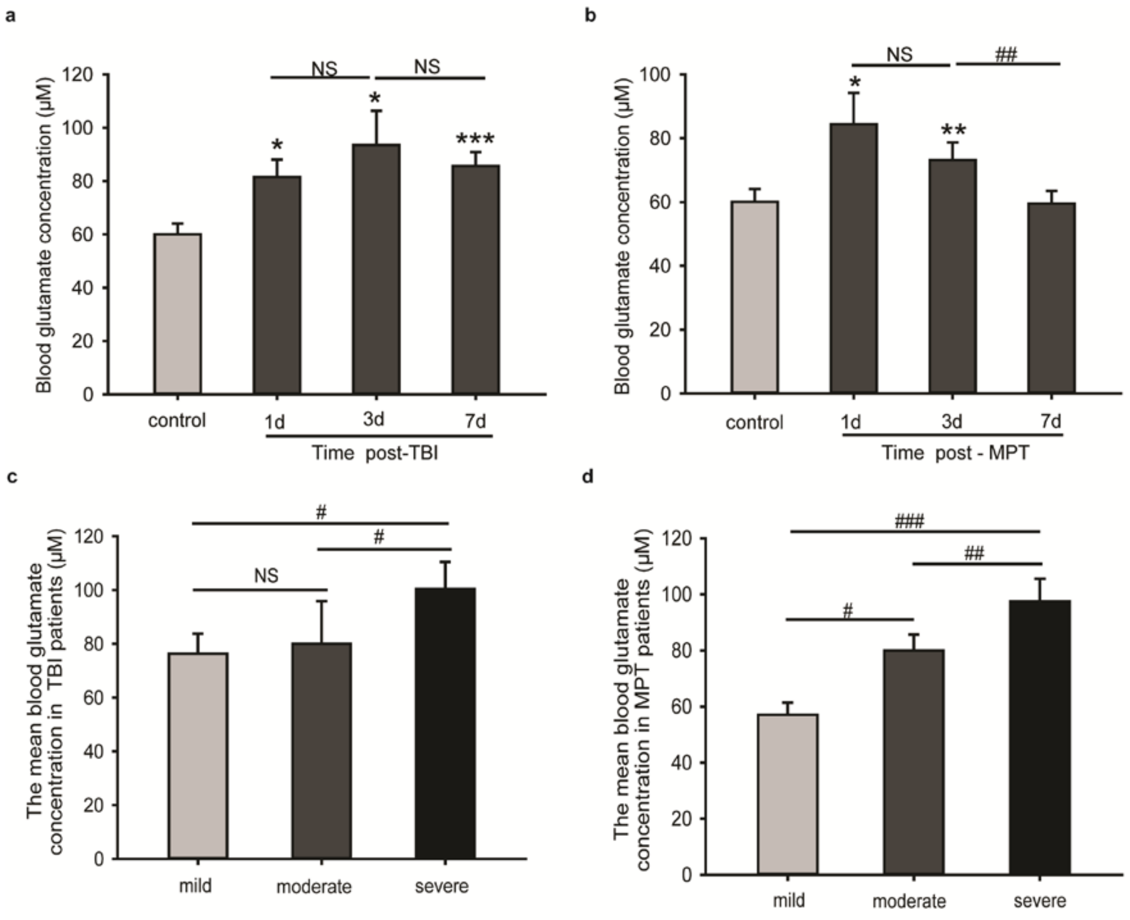

Figure S1. (a, b) Comparisons of blood glutamate concentrations on the 1st, 3rd and 7th day after injury in patients with TBI or MPT. **(c, d)** Comparisons of mean blood glutamate concentrations in patients with different severities of TBI or MPT. $^{*}p<0.05$, $^{**}p<0.01$, and $^{***}p<0.001$ compared to the control group; $^{\#}p<0.05$, $^{\#\#}p<0.01$, and $^{\#\#\#}p<0.001$ compared between the two groups; NS, not significant. Significance was determined by ANOVA with Tukey-Kramer *post hoc* tests or nonparametric Mann-Whitney U tests. MPT, multiple peripheral trauma; TBI, traumatic brain injury.

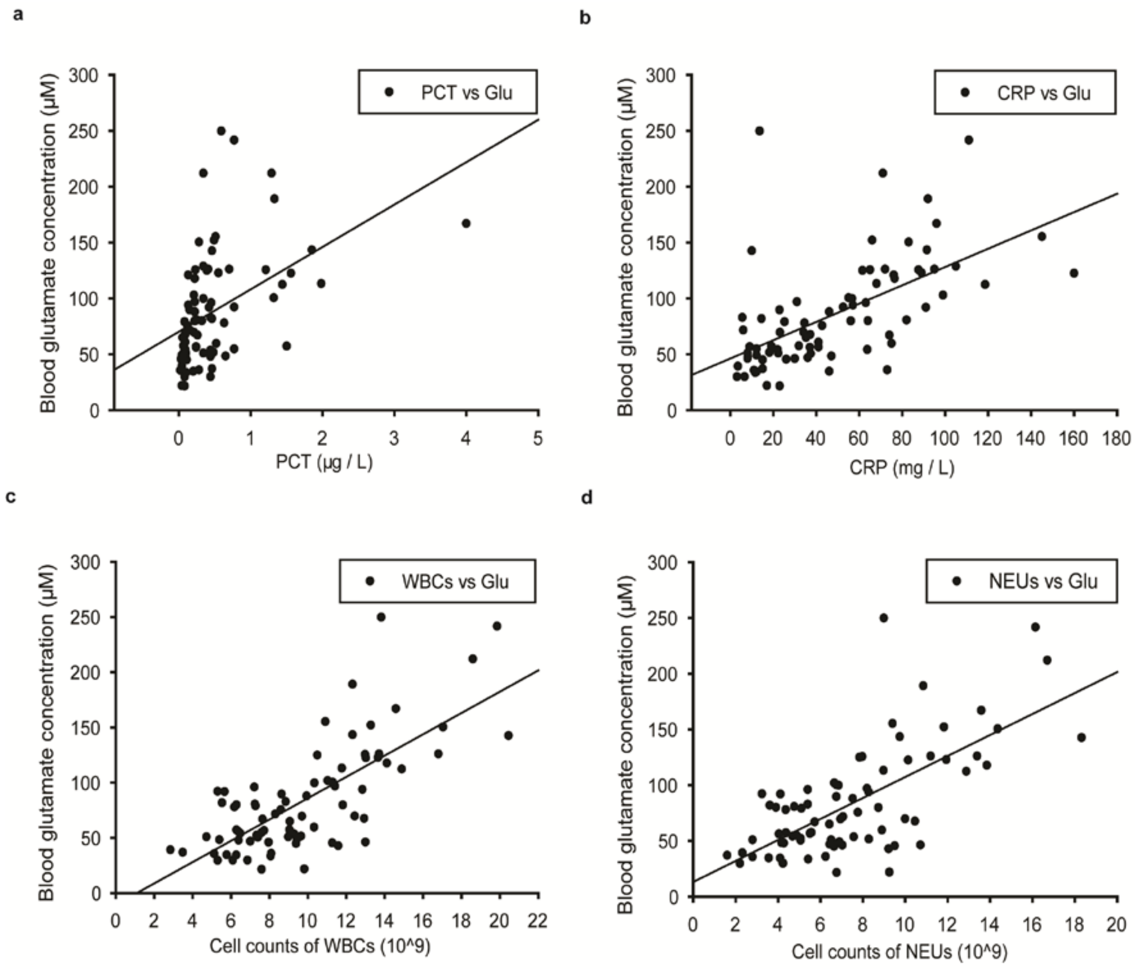

Figure S2. The Spearman's correlation test showed an obvious correlation between blood glutamate levels and inflammatory markers in patients with TBI-ALI (r=0.593, 0.670, 0.659, 0.596 for PCT **(a)**, CRP **(b)**, WBCs **(c)** and NEUs **(d)**, respectively; p=0.000). CRP, C-reactive protein; NEUs, neutrophils; PCT, procalcitonin; WBCs, white blood cells.
